# Cell Fate Reprogramming in the Era of Cancer Immunotherapy

**DOI:** 10.3389/fimmu.2021.714822

**Published:** 2021-07-21

**Authors:** Olga Zimmermannova, Inês Caiado, Alexandra G. Ferreira, Carlos-Filipe Pereira

**Affiliations:** ^1^ Cell Reprogramming in Hematopoiesis and Immunity Laboratory, Lund Stem Cell Center, Department of Molecular Medicine and Gene Therapy, Lund University, Lund, Sweden; ^2^ Wallenberg Center for Molecular Medicine, Lund University, Lund, Sweden; ^3^ Center for Neuroscience and Cell Biology, University of Coimbra, Coimbra, Portugal; ^4^ Doctoral Programme in Experimental Biology and Biomedicine, University of Coimbra, Coimbra, Portugal

**Keywords:** cancer immunotherapy, cellular reprogramming, tumor immunology, CAR-T, transcription factor, dendritic cell, antigen presentation, cancer vaccine

## Abstract

Advances in understanding how cancer cells interact with the immune system allowed the development of immunotherapeutic strategies, harnessing patients’ immune system to fight cancer. Dendritic cell-based vaccines are being explored to reactivate anti-tumor adaptive immunity. Immune checkpoint inhibitors and chimeric antigen receptor T-cells (CAR T) were however the main approaches that catapulted the therapeutic success of immunotherapy. Despite their success across a broad range of human cancers, many challenges remain for basic understanding and clinical progress as only a minority of patients benefit from immunotherapy. In addition, cellular immunotherapies face important limitations imposed by the availability and quality of immune cells isolated from donors. Cell fate reprogramming is offering interesting alternatives to meet these challenges. Induced pluripotent stem cell (iPSC) technology not only enables studying immune cell specification but also serves as a platform for the differentiation of a myriad of clinically useful immune cells including T-cells, NK cells, or monocytes at scale. Moreover, the utilization of iPSCs allows introduction of genetic modifications and generation of T/NK cells with enhanced anti-tumor properties. Immune cells, such as macrophages and dendritic cells, can also be generated by direct cellular reprogramming employing lineage-specific master regulators bypassing the pluripotent stage. Thus, the cellular reprogramming toolbox is now providing the means to address the potential of patient-tailored immune cell types for cancer immunotherapy. In parallel, development of viral vectors for gene delivery has opened the door for *in vivo* reprogramming in regenerative medicine, an elegant strategy circumventing the current limitations of *in vitro* cell manipulation. An analogous paradigm has been recently developed in cancer immunotherapy by the generation of CAR T-cells *in vivo*. These new ideas on endogenous reprogramming, cross-fertilized from the fields of regenerative medicine and gene therapy, are opening exciting avenues for direct modulation of immune or tumor cells *in situ*, widening our strategies to remove cancer immunotherapy roadblocks. Here, we review current strategies for cancer immunotherapy, summarize technologies for generation of immune cells by cell fate reprogramming as well as highlight the future potential of inducing these unique cell identities *in vivo*, providing new and exciting tools for the fast-paced field of cancer immunotherapy.

## Introduction

Cancer progression entails close crosstalk between tumor cells and the immune system. The evolution of cancer is driven by oncogenic mutations and the deregulation of important signaling pathways. The unleashed proliferative capacity leads to the accumulation of additional mutations, endowing tumor cells with selective advantages such as improved fitness and survival. Yet, such cancer diversity resulting from high mutational burden comes with a cost. The more mutations cancer cells acquire, the more likely they are recognized and eliminated by the immune system due to the expression of various tumor-associated antigens (TAAs) (1–3). However, the pressure imposed by immune surveillance further shapes cancer development, and the evolving tumor heterogeneity provides opportunities for the selection of clones that have developed mechanisms to evade the host’s immune system (4). These mechanisms include the downregulation of Major Histocompatibility Complex (MHC)-I molecules, over-expression of inhibitory checkpoints that prevent or attenuate T-cell responses, editing of neoantigens, or hijacking immune cells by creating an immunosuppressive environment. Overall, the complex, adaptable, and heterogeneous nature of cancer is characterized by its ability to disengage a variety of immune cells controlling specific modules of the immune response. For example, the tolerogenic environment established in some tumors promotes the activity of regulatory T-cells (Treg) and compromises dendritic cell (DC) function, impacting the productive induction of anti-tumor immunity (1, 5).

The idea of harnessing the body’s immune system to elicit anti-cancer responses has now stepped into the spotlight of cancer research. The field was pioneered by William Coley and the development of vaccines that activated anti-tumor immunity *via* exposure to bacterial toxins (6). The intravesical administration of Bacillus Calmette Guerain (BCG) in bladder cancer (7) and the use of interleukin (IL)-2 in renal cell carcinoma (8) foreshadowed the era of modern immunotherapy. Due to immense research efforts and broadened understanding of tumor biology and its interactions with the immune system, the past decade witnessed rapid development of cancer immunotherapy, resulting in several U.S. Food and Drug Administration (FDA) approvals, and the highlight of seminal discoveries leading to immune checkpoint blockade with a Nobel Prize. Immunotherapy changed the paradigm of cancer treatment, and it is now established as a potent therapeutic modality with long-term effects for multiple cancers, adding to the clinical toolbox of more conventional approaches encompassing chemotherapy, radiotherapy and surgery. However, only a minority of patients respond to immunotherapy, and some cancer types are not yet open to these treatments, emphasizing the need for new strategies to deliver clinical benefits to a larger number of patients and indications. Harnessing the potential of the immune cell repertoire and their functional features represent the next frontier in immunotherapy.

## Strategies for Cancer Immunotherapy

### Cancer Vaccines

The cancer vaccine concept relies on the induction of polyclonal T-cell-specific immune responses against multiple TAAs, offering a potential mechanism to target cancer heterogeneity. Multiple strategies have been employed to generate cancer vaccines, encompassing administration of irradiated whole tumor cells, DCs preloaded with TAAs, or *in vivo* antigen delivery *via* DNA or RNA (9). Therefore, successful cancer vaccination requires 1) a supply of purified and functional DCs to direct adaptive and innate immune responses and 2) a selection of highly immunogenic and specific TAAs to engage a powerful T-cell response exclusively against tumor cells (10).

DCs are phagocytic cells that can be found in peripheral blood, lymphoid organs, and all tissues. Given their exceptional ability to capture, process, and present antigens, as well as to produce cytokines and migrate to lymph nodes to activate T-cells, DCs play a pivotal role in orchestrating adaptive and innate immune responses against pathogens and cancer (11). Human DCs are a heterogeneous group comprising four main subtypes with distinct ontology, phenotype, and function – conventional type 1 (cDC1), conventional type 2 (cDC2), plasmacytoid (pDC), and monocyte-derived (mo-DC) DCs (12, 13). Conventional DCs are defined by the expression of CD11c and HLA class II and include two main subsets. cDC1 are developmentally dependent on BATF3 and IRF8 transcription factors and can be identified by surface expression of XCR1, CLEC9A, and CD141. They produce pro-inflammatory cytokines including IL-12, IL-29, and CXCL10 and their hallmark function is antigen cross-presentation and the generation of T helper (Th)1 response (14–16). In contrast, cDC2 are characterized by the expression of CD1c and constitute a highly heterogeneous subset that relies partially on IRF4 and Notch signaling. Some cDC2 govern type 2 responses against parasites, some sense extracellular pathogens, whilst others mount type 3 response driven by the activation of Th2/17 and Innate Lymphoid Cells (ILCs) (17, 18). In contrast, CD123+CD11c- pDC cells, dependent on transcription factor E2-2 and high levels of IRF8, are endowed with exceptional ability to sense viral and bacterial pathogens and release type I interferon in response to acute infection (19). CD1c+CD14+ mo-DCs are usually rapidly recruited during acute inflammation and are also capable of T-cell activation. However, compared to cells primed with conventional DCs that tend to launch effector-prone differentiation, mo-DCs preferentially drive differentiation of activated CD8+ T-cells into memory cells (20). While crosstalk and synergy among DC subsets are important for the complexity and magnitude of anti-tumor immune response (21), experiments in mice have implicated the presence and uncompromised function of cDC1 as vital to elicit efficient cancer immunity. As shown by Hildner et al., selective loss of cDC1 in BATF3^-/-^ mice leads to deficient cross-presentation and diminished CD8+ T-cell activation, resulting in deficient tumor control (22). In humans, genome-wide studies revealed that the presence of cDC1s within the tumor microenvironment correlates with a better outcome in patients (23). Given their role in orchestrating immunity, cDC1 activity is thus fundamental for effective response to immune checkpoint blockade or adoptive T-cell transfer (24–26).

**Figure 1:**
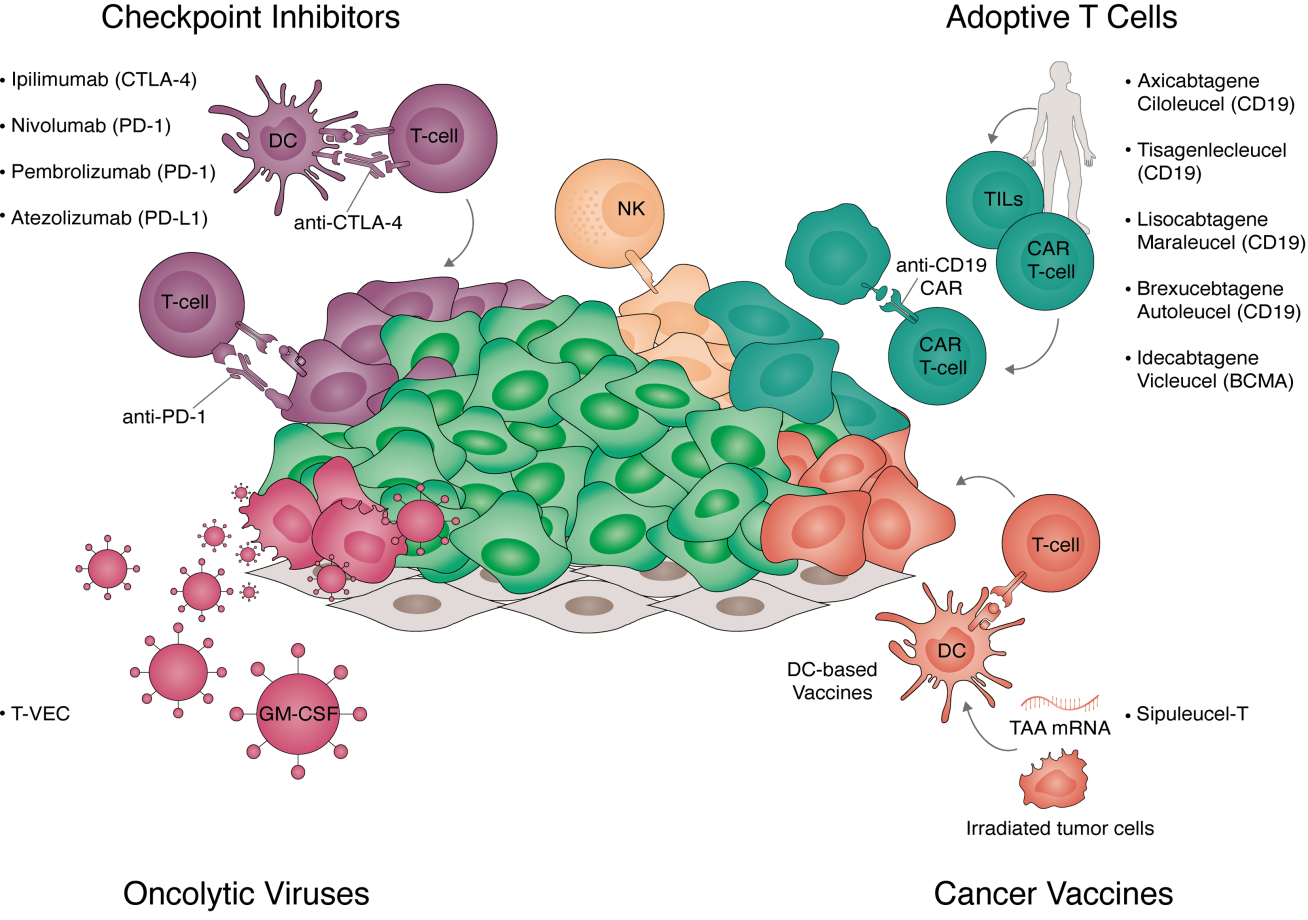
Strategies for cancer immunotherapy. Overview of the strategies for immunotherapy translated to clinical practice. Approved strategies include immune checkpoint inhibitors, adoptive T-cell therapy, cancer vaccines, and oncolytic viruses. Checkpoint inhibitors (top left panel) are antibodies targeting key negative regulators of T-cell activation. These include receptors expressed in T-cells targeted with anti-cytotoxic T-lymphocyte associated protein 4 (CTLA-4) (ipilimumab), anti-programmed cell death protein 1 (PD-1) (nivolumab, pembrolizumab) and receptors expressed in tumor cells targeted with anti-programmed death-ligand 1 (PD-L1) (atezolizumab). Adoptive T-cell transfer (top right panel) are autologous cellular therapies currently targeting hematological tumors. Approved Chimeric Antigen Receptor (CAR) T-cells target CD19 (axicabtagene ciloleucel, tisagenlecleucel, brexucabtagene autoleucel, and lisocabtagene maraleucel) or B-cell maturation antigen (BCMA) (idecabtagene vicleucel). Additionally, tumor infiltrated lymphocytes (TILs) expanded *ex vivo* and natural killer (NK) cells are currently in clinical trials. Cancer vaccines (bottom right panel) exploit the antigen presentation capacity of dendritic cells (DC) to trigger polyclonal T-cell responses to tumor-associated antigens (TAA). These include irradiated tumor cells expressing granulocyte-macrophage colony-stimulating factor (GM-CSF, GVAX), DCs loaded with tumor antigens (sipuleucel-T), or the delivery of mRNA *in vivo* encoding multiple TAAs. Oncolytic viruses (bottom left panel) target cancer cells own vulnerability to viral infections while inducing immunogenic cell death. Talimogene laherparepvec (T-VEC) is an approved intra-tumoral therapy consisting of a type-1 herpes virus expressing GM-CSF.

Despite considerable efforts, the clinical translation of cancer vaccines into potent therapies has been challenging. Traditional DC vaccines are mostly based on DCs isolated from patients, or on DCs differentiated from autologous peripheral monocytes, which are further loaded with TAAs in tumor lysates. Spiluleucel-T is the only FDA-approved vaccine that targets the PAP antigen and increases overall survival in hormone-refractory prostate carcinoma by approximately 4 months (27) (**Figure 1**). However, mo-DCs have reduced capacity for efficient antigen presentation, compromising the efficiency of DC vaccines in most patients (20). An interesting approach may lie in cancer vaccines utilizing purified DC subsets. Importantly, vaccines derived from allogenic pDCs primed cytotoxic immune responses in advanced melanoma patients (28). Similarly, autologous cDC2 cells mounted CD8+ T-cell responses and delivered long-term progression-free responses in approximately 25% of melanoma patients (29). Combining DC subsets may be another interesting strategy to enhance the efficiency of DC vaccines as illustrated by pDC and cDC2 cooperation. pDCs produce high levels of CXCL3 that attract a variety of immune effectors, including CD8+ T-cells, CD56+ T-cells, and γδ T-cells, while cDC2 secure sufficient antigen presentation and T-cell priming (30). However, cancer vaccination strategies would potentially benefit if the preparation of vaccines could be done from pure, functional cDC1s, currently limited by their rarity. In this regard, identification of transcription factors imposing the identity of multiple DC subtypes by cellular reprogramming may open the door for the generation of pure populations of DCs (31).

Despite the importance of using potent antigen-presenting cells, not all types of cancer vaccines include DCs directly. The development of GVAX is based on the use of irradiated tumor cells expressing GM-CSF which further modulate antigen presentation and support DC function (32).

Therapeutics based on nucleic acids have also emerged as an alternative to conventional vaccine approaches. mRNA vaccines recently attracted enormous attention as the basis of COVID-19 vaccination. In cancer immunotherapy, the mRNA approach has been explored for delivery of TAAs directly to DCs, offering an elegant solution for cancer vaccination. mRNA vaccines are delivered by lipid nanoparticles containing lipoplexed RNA molecules encoding selected neoantigens or TAAs (33). Promising results were recently reported by interim results of phase 1 clinical trial of FixVac, an RNA-LPX vaccine targeting four highly prevalent melanoma-associated antigens that are shared by most patients. When administered in a multiple-dose regimen, FixVac alone, or in combination therapy with checkpoint inhibitors, induced strong CD4+ and CD8+ immune responses and delivered durable responses in patients with unresectable melanoma (34). Nevertheless, a careful selection of suitable treatment combinations, as well identifications of biomarkers predicting response to cancer vaccines may help to maximize the clinical benefit of the vaccination strategy for a broader group of patients (35).

### Oncolytic Viruses

Tumor cells frequently accumulate mutations in pathways securing cellular anti-viral defense, rendering themselves vulnerable to viral infections. This cancer vulnerability has been also exploited as a potential strategy to control tumor growth. Oncolytic viruses (OVs) are engineered to selectively infect and replicate in tumor cells and to induce immunogenic death. Hypothetically, the administration of OV therapy should be able to target natural cancer-immunity interactions at multiple levels: OV-mediated lysis of tumor cells promotes the release of TAAs and type I interferon, boosting anti-tumor immunity.

Since the first report of conditionally replicating Herpes Simplex type 1 (HSV-1) engineered virus which prolonged survival in nude mice with glioma (36), a myriad of viral backbones has been exploited, encompassing adenoviruses, measles, coxsackie, polio, reoviruses, or poxviruses. The selective tropism for tumor cells can be modulated by genetic modifications of the capsid, utilizing epitopes targeting receptors exclusively or highly expressed on tumor cells (37).

Furthermore, the engineering of OVs allows enforced expression of genes with therapeutic potential, including TAAs, costimulatory molecules, and cytokines (e.g. GM-CSF, Il-2, IL-12, or TNFα), which can further amplify immune responses and counteract tumor-induced immunosuppression. The only FDA-approved oncolytic viral-based therapy is talimogene laherparepvec (T-VEC), used in advanced melanoma patients (**Figure 1**). T-VEC is an attenuated HSV-1 virus harboring a deletion of neurovirulence genes, engineered to express GM-CSF to promote T-cell priming and boost immunogenicity. In advanced melanoma patients, T-VEC achieved durable responses and prolonged survival in nearly 20% of patients, benefiting particularly those at early stages of metastasized disease (38).

Given their unique characteristics, OVs are promising therapeutic agents, but their efficacy, when used as a single therapy, leaves room for improvement. In this way, the versatility of the oncolytic viral platform makes it suitable for combination with other immunotherapies and conventional treatments. The combination of T-VEC with immune checkpoint inhibitors showed improved efficacy in melanoma without an increase in adverse effects, delivering long-term response in up to 62% of patients (39–41). The effect is attributed to the ability of OVs to convert immunologically cold tumors into immune hotspots, overcoming systemic immunosuppression (42).

### Immune Checkpoint Inhibitors

Immune checkpoints were originally discovered as a group of evolutionarily conserved molecules acting as negative regulators of T-cell activation, with cytotoxic T-lymphocyte Antigen 4 (CTLA-4) and PD-1 inhibitors being recently approved for clinical practice. CTLA-4 is primarily found on T-cells and controls early-stage T-cell activation by counteracting the activity of the co-stimulatory molecule CD28 (43–45). Unlike CTLA-4, PD-1 molecule is expressed following T-cell activation, and its major role is to tame T-cell activity in peripheral tissues, preventing tissue damage. As their natural role lies in the maintenance of self-tolerance, and modulation of the duration and magnitude of physiologic immune responses (46, 47), the engagement of immune checkpoints is one of the mechanisms which cancer cells deploy to hijack the host immune defense. Therefore, the expression of PD-1 ligand (PD-L1) was identified on many tumor cells including hematologic malignancies, glioblastoma, lung, kidney, prostate, breast, and ovarian carcinoma, and has a critical role in suppressing immune-surveillance mechanisms (48). Interestingly, within the tumor microenvironment, PD-L1 can be expressed by myeloid cells including macrophages and DCs. The deletion of PD-L1 in the DC compartment, but not in macrophages, strengthened cytotoxic CD8+ mediated responses and abrogated tumor growth (49).

Inhibition of immune checkpoint receptors employs synthesized antibodies as a therapeutic intervention disrupting the ability of some cancer cells to evade a patient´s immune response (45). Administration of anti-CTLA-4 (ipilimumab) prolongs survival in patients with metastatic melanoma where conventional treatment approaches failed (50). Notably, ipilimumab was the first FDA-approved checkpoint inhibitor, followed by anti-PD-1 inhibitors (nivolumab and pembrolizumab) for the treatment of squamous non-small cell lung carcinoma (NSCLC) as well as melanoma (51, 52) (**Figure 1**). Given their impact in reactivating anti-tumor immunity in humans, checkpoint inhibitors became a milestone achievement in cancer research of the past decade, as highlighted by the Nobel Prize awarded to James Allison and Tasuko Honjo in 2018.

Despite delivering unprecedented long-lasting anti-tumor responses (53), persisting for more than 10 years after discontinuation of therapy in some patients, important challenges remain: 1) only about 20% of patients benefit from immune checkpoint treatment (54) and, 2) cancer types that build a local strong immunosuppressive environment do not respond consistently to these therapies (55), suggesting that the future lies in combining interventions targeting mechanistically independent immune evasion mechanisms.

Following the therapeutic success in metastatic melanoma and NSCLC, checkpoint inhibitors became increasingly used as a treatment alternative in a range of solid tumors, particularly in cancers attributed to carcinogen exposure or infectious origin. In addition to NSCLC, smoking is associated with the development of bladder cancer, head and neck carcinoma, renal cell carcinoma, or stomach malignancies, conferring a high tumor mutational burden (56–60). Importantly, as high mutational load generally predicts a good response to checkpoint inhibition (61), patients with mismatch repair deficiency (MMR) are suitable candidates for checkpoint immunotherapy (62). Recent publications also reported therapeutic success of checkpoint inhibition in Hodgkin lymphoma (63), and pembrolizumab and anti PD-L1 (atezolizumab) are currently setting the way to improve the outcome in triple-negative breast carcinoma, as PD-L1 positive patients reached 25-month survival compared to 18 months in the control group (64). Overall, the efficacy of checkpoint inhibitors as an adjuvant therapy complementing surgery or chemotherapy is currently being evaluated in several clinical trials (65). On the other hand, the administration of checkpoint inhibitors is associated with excessive immune activation, with almost every third patient suffering from immune-related adverse effects, encompassing diarrhea, colitis, hepatitis, development of hypothyroidism, type 1 diabetes, or adrenocortical insufficiency (66). Notably, melanoma patients who received combined CTLA-4/PD-1 inhibition showed longer overall survival although at the cost of higher adverse effects (53, 67). Although the clinical benefit clearly outweighs toxicity, additional efforts should be made towards strategies to identify patients who can benefit the most from checkpoint therapy.

### Adoptive Cellular Therapy

Harnessing T-cells for immunotherapy has long been of prime interest to cancer research. T-cells are unique immune cells: they are long-lived in resting conditions and proliferate extensively when encountering specific stimuli. T-cells elicit specific, robust, and enduring responses against tumor cells. As their action is systemic, they can reduce the tumor burden at the primary site and eradicate distant metastatic sites. On the downside, the manufacturing of lymphocytes for immunotherapy is challenging as it is so far tailored for each individual patient.

#### Tumor-Infiltrating Lymphocytes

First attempts to use T-cells in immunotherapy were based on tumor-infiltrating lymphocytes (TILs), a pool of T-lymphocytes pre-trained to recognize patient´s specific cancer cells. When expanded, pre-activated *ex vivo*, and reinfused into patients, TILs induce rapid tumor regression (68, 69). In the context of melanoma, around 20% of patients show durable responses to administration of *ex vivo* expanded TILs (5-year survival 93%) (70). Despite achieving outstanding responses in melanoma, the nature of the therapy makes it amenable only for highly mutated, immunologically hot tumors. Indeed, TIL-based therapies entered clinical trials also for the treatment of head and neck cancer, cervical carcinoma, ovarian carcinoma, or NSCLC.

#### CAR T-Cells

Chimeric antigen receptors (CAR) T-cells represent another breakthrough in immunotherapy. CAR T-cells are genetically engineered to express a synthetic T-cell receptor tailored to recognize surface antigens on cancer cells. The current generations of chimeric receptors usually consist of a single-chain variable fragment portion of the target receptor, along with the costimulatory intracellular domain of CD28, 4-1BB, or OX40 (71). CD19 targeting CAR T-cell-based immunotherapy encountered massive success in the treatment of B-cell hematologic malignancies, which translated into FDA approval of axicabtagene ciloleucel for relapsed or refractory large B cell lymphoma, as well as tisagenleucel in refractory pediatric B-cell acute lymphoblastic leukemia and B-cell lymphoma. More recently, B-cell maturation antigen (BCMA) CAR T-cells (idecabtagene vicleucel) were approved for therapy of advanced myeloma (72, 73), brexucabtagene autoleucel for mantle cell lymphoma, and lisocabtagene maraleucel for relapsed or refractory large B-cell lymphoma (**Figure 1**). However, the high efficacy of CAR T-cell treatments also comes with relatively high toxicity and particularly, with the risk of cytokine release syndrome (CRS) (74).

Despite achieving tremendous efficiency in the treatment of hematological malignancies, clinical success with CAR T-cells in the context of solid tumors is less encouraging, having hit multiple roadblocks from a limited number of targetable antigens to the inefficient trafficking of CAR T-cells into the tumor sites. Nevertheless, attempts are being made with CAR T-cells armed against ERBB2/HER2 receptor tyrosine kinase frequently over-expressed by tumor cells, as well as targeting TAAs including mesothelin, carcinoembryonic antigen (CEA), GD2 (75–79), or IL13Ra2 (80).

#### NK Cells

NK cells are innate cytotoxic lymphocytes critical for defense against abnormal cells, and, therefore, attracted plenty of attention in cancer immunotherapy. Unlike T-cells, their activation is induced *via* the direct interaction of native NK receptors with ligands on target cells. They are specially competent in recognizing cells that have lost MHC-I expression (81, 82), and lyse abnormal cells by granzyme B and perforin. NK cells also secrete a myriad of cytokines and chemokines, including IFN-γ, TNFα, IL-13, CCL3, CCL4, and GM-CSF, and, therefore, can modulate anti-tumor response by influencing trafficking other immune cells including cDC1s (83). However, NK cell function is severely compromised in patients, mostly due to the reduced expression of NK-activating ligands and suppressive effect of TGF-β within the tumor microenvironment (84–86), ultimately leading to their inability to lyse cancer cells and contributing to the progression of tumor development.

Adoptive NK cell therapy was originally successful in the treatment of hematologic malignancies, as administration of alloreactive NK cells elicited graft versus leukemia response and protected bone marrow transplanted acute myeloid leukemia (AML) patients from leukemia relapse (87), which largely prompted clinical endeavors in the application of NK transfer for solid tumors treatment.

In addition to AML, NK cell immunotherapy is delivering promising results also in children with neuroblastoma who were treated with anti-GD2 followed by allogenic NK transfer, eliciting a response in approximately 40% of patients (88). An exciting treatment opportunity further opens with the generation of NK cells expressing CARs – which are currently being tested, for example in glioblastoma (HER2-NK-92), pancreatic carcinoma (ROBO1-NK), colorectal carcinoma (Epcam-NK), or in patients with relapsed or refractory AML (CD33-NK-92) (89–91). As NK mediated cytotoxic happens independently on antigen processing, the use of NK cells may help overcome immunotherapy roadblocks imposed by lost antigen presentation or in tumors with a low mutational burden. NK therapy bypasses the need of the HLA-matched donors (92), and holds promise as off-the-shelf, bio-banked immunotherapy, which could be available on demand. Isolation of NK cells from donors at scale is problematic, but recent efforts have been made towards the manufacturing of NK cells *via* directed differentiation of induced Pluripotent Stem Cells (iPSCs), paving the way to harness cell reprogramming for immunotherapy (93, 94).

## Immune Cell Programming and Reprogramming

The definition of individual immune cellular identities entails unique transcriptional, epigenetic, and functional signatures. Advances in next-generation sequencing and single-cell technologies have revealed additional layers of complexity in cell identity and cellular differentiation. For many years, cell differentiation was perceived as an irreversible, one-way road, metaphorically depicted as balls rolling down hills. Pioneering work with somatic cell nuclear transfer in frogs by John Gurdon in 1962 demonstrated that nuclei of terminally differentiated somatic cells retain the genetic information necessary to generate an entire individual, laying the foundation of cellular reprogramming (95). Decades later, Ian Wilmut cloned sheep from an adult mammary gland cell nucleus, providing evidence that somatic cell reprogramming can be achieved from adult cells in mammals (96). Discoveries with somatic cell nuclear transfer then prompted efforts to identify the factors that elicit reprogramming. The landmark discovery of Shinya Yamanaka in 2006, showing that enforced expression of the transcription factors OCT4, SOX2, KLF4, and MYC induced pluripotency in fibroblasts (97, 98) created a sea change in stem cell research.

The capacity to modify cell identity with a small set of transcription factors created exciting opportunities to investigate the fundamental principles of cell identity, provided a strategy to generate rare subtypes for drug screening and disease modeling, and opened avenues in regenerative medicine and cell replacement therapy.

Reprogramming to pluripotency was successfully shown from multiple somatic cell populations including fibroblasts, keratinocytes, neural progenitors, and peripheral blood cells (97–102), demonstrating the feasibility of iPSC generation from patient´s own biological material. iPSC reprogramming can generate target cell types on a large scale suitable for *ex vivo* production, as well as enable *in vitro* engineering to deliver desirable genetic modifications (e.g. CAR). However, clinical exploitation of iPSC technology has several major challenges.

iPSCs, just like embryonic stem cells (ESCs), are pluripotent and retain the capacity to generate all cell types of the embryo when provided with stage-specific signaling allowing differentiation to the cell type of interest. It is however a major challenge to fully recapitulate this process *in vitro*. In addition, the iPSC differentiation process is very dependent on line-to-line variability, and generates a heterogeneous population of cells, hampering clinical translation. Importantly, despite epigenetic resetting during induction of pluripotency, iPSCs may retain residual parental epigenetic memory which may bias their differentiation potential into certain cell types based on the source of donor cells used to establish iPSC (103). There is also the additional safety concern due to possibly contaminating iPSCs in the differentiated cell population as iPSCs are pluripotent and may generate teratomas *in vivo.* Therefore, to meet safety standards for clinical trials, more stringent protocols to purify differentiated cells are currently being developed. For example, a study exploiting iPSCs as a therapy of Parkinson disease is using a positive selection of differentiated dopaminergic neurons with anti-chorin antibody (104), while another clinical trial evaluating iPSCs in heart failure treatment uses the elimination of undifferentiated CD30+ cells by an antibody conjugated with an antimitotic agent (105).

**Figure 2:**
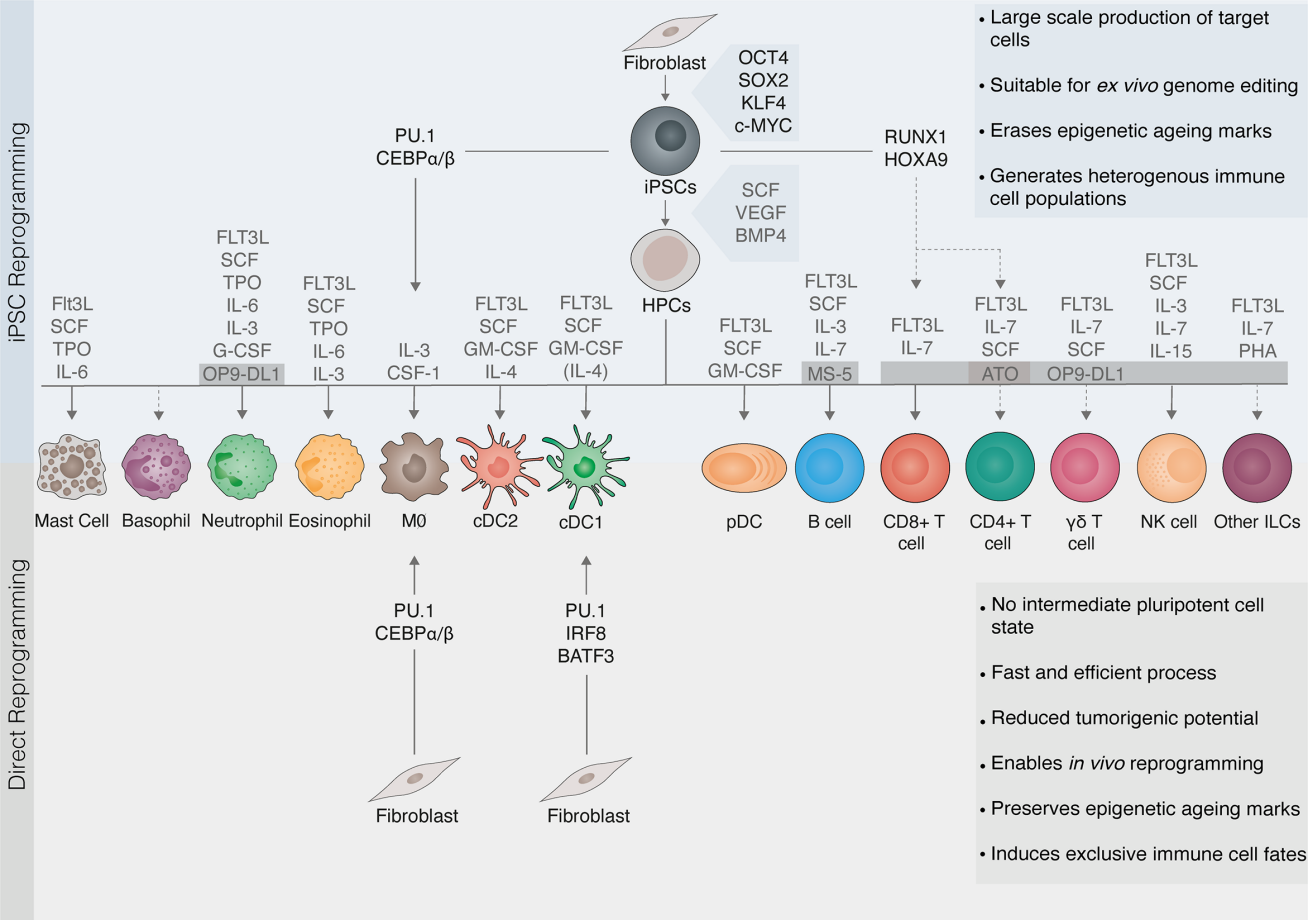
Cell reprogramming as a source of immune cells. Cellular reprogramming provides an opportunity to generate tailored immune cells for immunotherapy. Somatic cells can be reprogrammed to induced pluripotent stem cells (iPSCs) with the transcription factors OCT4, SOX2, KLF4, and c-MYC. iPSCs are then differentiated to hematopoietic progenitor cells (HPCs) with the cytokines stem cell factor (SCF), vascular endothelial growth factor (VEGF), and bone morphogenetic protein 4 (BMP4). Protocols for the differentiation of HPCs into myeloid and lymphoid lineages involve exposing cells to complex cytokine cocktails and/or feeder layers including MS-5, OP9-DL1, or artificial thymic organoids (ATO) supporting lymphopoiesis. Due to the self-renewal capacity of pluripotent stem cells, iPSC reprogramming (top panel) can generate immune cells on a large scale and is suitable for *ex vivo* genome editing. Cells undergo rejuvenation and erasure of epigenetic marks associated with aging or exhaustion. However, iPSC differentiation into mature immune cells is challenging (dashed lines) and leads to the generation of heterogeneous populations. Direct reprogramming (also known as transdifferentiation, bottom panel) refers to a change in cell fate mediated by lineage transcription factors that, unlike in iPSC reprogramming, does not involve pluripotent intermediates. The direct reprogramming of fibroblasts to macrophages (MØ) or conventional dendritic cells type 1 (cDC1) induce exclusively the target immune cell fate. Direct reprogramming is a faster and more efficient process. It can be elicited both *in vitro* and within the target tissue, thus having potential for *in vivo* reprogramming. It also reduces tumorigenic risks from contaminating stem cells. Moreover, direct reprogramming may retain epigenetic aging hallmarks, making it more suitable for modeling aging-related disease but more difficult to achieve complete epigenetic reprogramming. Transcription factors can also be applied to accelerate and direct the differentiation of iPSCs (also known as forward reprogramming, top panel). FLT3L, Fms-like tyrosine kinase 3 ligand; TPO, Thrombopoietin; IL, interleukin; G-CSF, granulocyte colony-stimulating factor; GM-CSF, granulocyte-macrophage colony-stimulating factor; cDC2, conventional dendritic cells type 2 DCs; pDCs, plasmacytoid DCs; NK, natural killer; ILCs, innate lymphoid cells.

iPSC technology holds the potential to provide a source of a wide range of blood cells. Differentiation of pluripotent stem cells recapitulates events known to occur during embryonic development. Given the complexity of hematopoiesis, which encompasses various anatomic locations and the formation of progenitors *via* endothelial-to-hematopoietic transition in several waves, *in vitro* differentiation of hematopoietic precursors then requires multiple steps involving the formation of embryonic bodies allowing mesodermal development. Furthermore, a range of differentiation cytokine cocktails, as well as co-culture with feeder layers promote cell growth and differentiation (**Figure 2**). Since the usage of multiple feeder cells is self-limiting for expansibility of target cell production, the development of feeder-free differentiation techniques is critical for wide-scale translation.

The possibility of reverting somatic cells into the pluripotency encouraged investigation of combinatorial codes driving the conversion of somatic cells into another unrelated somatic cell type directly, without transiting through intermediate pluripotent or multipotent stages. Bypassing developmental stages, direct cellular reprogramming is specific, more efficient, faster, and easier from the manufacturing point of view, as well as suitable for *in vivo* applications as it does not impose the risk of malignant transformation associated with pluripotency. On the downside, cells generated *via* direct reprogramming may show premature phenotypes and higher infidelity to their physiologic counterparts caused mainly by the incomplete epigenetic memory loss during the reprogramming process. For instance, while both iPSC-derived and directly reprogrammed neurons launch gene regulatory networks specific for neurons, directly reprogrammed cells retain basal levels of the parental identity attributed to the incomplete erasure of DNA methylation and histone marks of the parental genome (106).

Nevertheless, the number of cell types generated *via* direct cellular reprogramming is rapidly increasing. The first example of direct reprogramming was in 1987, with the reprogramming of fibroblasts to myoblasts *via* forced expression of a single transcription factor, MyoD1 (107). The combinatorial approach has enabled the identification of master lineage transcription factors for the direct reprogramming into neurons, cardiomyocytes, blood progenitors, hepatocytes, neurons, Sertolli cells, adipocytes, chondrocytes, osteoblasts, or pancreatic insulin-producing β-cells (108–120).

Overall, a better understanding of cell fate plasticity opened new avenues for the potential application of cell reprogramming in regenerative medicine and tissue repair. Importantly, cell reprogramming is now providing the means to modulate tumor immunity either by iPSCs immunomodulatory properties or through the generation of discrete immune cell populations that can reinitiate the cancer-immunity cycle. Cell reprogramming may facilitate large-scale and standardized manufacturing of clinically relevant cell types which can be bio-banked and used as an off-the-shelf therapy. Such approach could meet limitations of current strategies for cancer immunotherapy and provide alternative sources of important immune players such as T-cells, NK-cells, or DCs, addressing current challenges of limited availability, and functional exhaustion.

### iPSCs in Development of Cancer Vaccines

Given their proliferative potential and metabolic requirements, iPSCs share many features with cancer stem cells. Among these, iPSCs express TAAs which may be recognized by the immune system (121, 122). Interestingly, immune responses launched by iPSCs do not propagate the development of autoimmunity, indicating that fetal-like and adult settings are associated with a differential expression of antigens. To this end, their immunogenic properties render iPSCs as an interesting, patient-specific, source of cells for manufacturing prophylactic cancer vaccines, circumventing tumor mass collection. The efficacy of iPSCs-based vaccines has been addressed. Li et al. compared the efficiency of immunization with iPSCs and ESC lines in a mouse colorectal cancer model and demonstrated that while both vaccines activated IFN-γ and IL-4 producing T-cells, tumor growth was prevented only in the context of ESC-based vaccine (123). In contrast, transplantation of mouse fibroblast-derived iPSC into teratoma-bearing mice leads to T-cell infiltration and tumor shrinkage (124). More recently, iPSC-based vaccination halted growth of murine breast cancer, mesothelioma, and melanoma, as well as prevented melanoma relapse (122).

### Reprogramming Into Macrophages

Macrophages are malleable immune cells that promptly respond to environmental cues and signals. Macrophages are essential for tissue homeostasis by clearing dying cells and invading pathogens, but their role is ambiguous and under certain conditions, macrophages actively contribute to the development of atherosclerosis or neurodegeneration. The ambiguity of macrophage functions is given by their polarization towards the promotion or suppression of immunity. In the context of cancer, pro-inflammatory macrophages (M1) can contribute to tumor clearance by the production of IL-12 and other pro-inflammatory cytokines, generation of reactive oxygen species, and presentation of antigens from engulfed dead tumor cells. However, cues produced by tumor cells can polarize macrophages into M2 anti-inflammatory phenotype, inducing an immunosuppressive microenvironment and supporting tumor development (125).

Several publications reported successful differentiation of macrophages from iPSCs upon culture with IL-3 and M-CSF (126, 127). iPSC-derived macrophages displayed a potent phagocytic capacity and rescued immunodeficient mice from Pseudomonas aeruginosa infection (126). Despite displaying functional features, differentiated macrophages showed low polarized phenotypes, expressing features of both pro-inflammatory M1 (CD195+, HLA-DR+) and suppressive M2 macrophages (CD163+, CD206+). Similarly, this ambiguity was mirrored by secretion of both pro-inflammatory cytokines, such as IL-6, CXCL8, CCL2, CXCL10, and M2-like cytokines, IL-10 and VEGF (128). A major challenge for the derivation of immune cells from iPSC resides in the specification of definitive hematopoietic stem cells (HSCs). Despite intense efforts in the last two decades, there is still no robust protocol for the generation of engraftable HSCs from iPSCs that would provide a renewable source of definite hematopoietic and immune cells (129). However, in contrast to other immune cells, microglia, the only lifelong resident immune cells of the central nervous system (CNS), have been suggested to be derived predominantly from yolk sac progenitors (130). This increases the feasibility of generating these highly specialized macrophages with fidelity from iPSCs that have recognized roles in neurodegenerative diseases (131–133).

Microglia can cross the blood-brain barrier, which makes them interesting vehicles for the delivery of therapeutic agents to the CNS. To this end, macrophages can be armed with a single chain variable fragments (scFv) specific to β-amyloid to help devour amyloid plaques, bringing new potential for the treatment of Alzheimer’s disease (134). A similar approach of macrophage modification can be also used to enhance the macrophage activity against certain tumors. As an example, macrophages generated from iPSCs engineered to express CD20-specific scFv eliminated B-lymphoblastic leukemic cells both *in vitro* and in a xenograft murine model (134). Macrophages modified with CARs to direct the engulfment of specific antigens have also been produced, showing an increased ability to phagocyte cancer cells (135).

Macrophages can also be generated by direct cellular reprogramming (**Figure 2**), *via* combined expression of the transcription factor CEBPα or CEBPβ, which favors myeloid development by the downregulation of B-cell commitment transcriptional factor PAX5, and PU.1 which is critical for instructing myeloid transcriptional program (136). By this approach, human fibroblasts were successfully reprogrammed into functional macrophage-like cells in less than a week, acquiring the ability to phagocyte bacteria and mount inflammatory responses (137). Transcription factors can also be applied to accelerate and direct the differentiation of iPSCs, a process also known as forward reprogramming (138, 139). Chen et al. have recently tested this strategy for the induction of immune cells. PU.1 and CEBPα were utilized to orchestrate rapid reprogramming of iPSCs into induced microglia endowed with several functional properties including LPS/IFN-γ-induced inflammatory responses, ADP/ATP-evoked migration, and phagocytic capacity (140). These findings highlight the advantage of employing direct reprogramming core regulatory circuits not only to define immune cellular identities but also to support their generation from somatic or pluripotent cells.

### Reprogramming Into Dendritic Cells

DCs are key orchestrators of both innate and adaptive immunity. Given the low abundance of DCs in peripheral blood, cell reprogramming may offer an exciting opportunity to generate DC subsets at scale. The protocols to differentiate DC subsets from iPSCs are at their infancy, revealing heterogeneity of generated DC populations as one of the main challenges to overcome. DCs derived from iPSCs need multistep protocols, including differentiation with FLT3L, SCF, GM-CSF (that enrich in pDC-like cells), and IL-4 (enriching in cDC1 and cDC2-like cells), as well as the maturation signals TNFα and LPS (141).

Sachamitr et al. reported the directed differentiation of iPSCs into CD141+ cDC1 cells resembling naturally occurring cDC1s. Consistently with their capacity for antigen presentation, CD141+ iPSC-DCs acquired immunostimulatory CD40+ CD86+ phenotype upon TLR engagement and could elicit activation and proliferation of naïve T-cells. However, induced DCs produced only limited amounts of IL-12 cytokine and in a steady state displayed tolerogenic features characteristic for tissue-resident DCs (142). CD141+ DC1-like cells were differentiated also from iPSCs derived from T-cells and showed functional capacity to process antigens (143).

Additionally, iPSCs derived DCs displayed rather fetal, immature phenotypes, reflected by the low expression of HLA class II and costimulatory molecules, low secretion of the hallmark cytokine IL-12, and limited ability to activate antigen-specific cytotoxic T-cells *via* antigen cross-presentation (134). Nevertheless, immature fetal DC phenotype can be partially circumvented by differentiation of iPSCs generated from CD11c+ DCs, resulting in an overall improvement of DC-specific functions and a significant increase in DC-mediated T-cell activation (144). Interestingly, the utilization of the iPSC reprogramming platform also allows to introduce genetic modifications which can potentiate anti-tumor responses. For instance, vaccination with mo-DC-like DCs derived from modified, CEA expressing iPSCs restricted growth of CEA-positive murine colon carcinoma model (145).

We have recently reported the generation of cDC1-like cells by direct cellular reprogramming from mouse fibroblasts with the master regulators PU.1, IRF8, and BATF3 (**Figure 2**). Only nine days after reprogramming initiation, induced DCs established a cDC1 specific transcriptional program and morphology resembling their natural counterparts. Importantly, induced DCs were endowed with cDC1 hallmark functional properties including secretion of IL-12 and other pro-inflammatory cytokines, phagocytosis of dead cells, and cross-presentation (146). This study opens the way to employ direct cell reprogramming and forward reprogramming principles for the specification of homogeneous sub-populations of DCs.

### Reprogramming Into Granulocytes

In contrast with numerous efforts towards reprogramming into macrophages and dendritic cells, differentiation towards granulocytic lineages was investigated less intensively. However, generation of autologous granulocytes holds promise to replenish immune competence in patients after HSC transplantation or chemotherapy to prevent the development of sepsis and severe bacterial infections. Using multistep protocols and growth factor cocktails, granulocytes have been successfully differentiated from iPSCs. iPSCs are first instructed towards hematopoietic precursors, followed by the induction of myeloid lineage and subsequent terminal differentiation towards granulocytic fate with FLT3L, SCF, TPO, IL-3, IL-6, G-CSF. This results in the generation of enriched populations of eosinophils, and upon OP9-DL1 co-culture, allows the specification of neutrophils (126, 147, 148). In accordance with their natural counterparts, iPSC-derived granulocytes acquired competence to phagocyte, produce reactive oxygen species, and, to a lesser extent, the ability to migrate in response to chemotactic stimuli (147). Alike many other immune cells, the instructor transcription factors that in combination impose granulocytic cell identity remain to be identified.

### Reprogramming Into NK Cells

NK-based immunotherapy recently received plenty of interest. Many clinical and preclinical trials demonstrated significant efficacy of NKs particularly against AML and ovarian cancer (149). However, current NK-based immunotherapy products face hurdles imposed mainly by relatively high variability in the efficacy of NKs derived from different donors. Furthermore, allogenic NK products are usually prepared from donor blood by apheretic depletion of CD3 and CD19 cells, resulting in a monocyte/NK cell mixture containing approximately only 30-40% of NK cells in the final product (150). Therefore, immunotherapy approaches may leverage iPSCs to generate an off-the-shelf supply of NK cells with a known haplotype in a highly standardized manner. Indeed, iPSC-derived NK cells are currently being tested in the clinical setting, representing the first example of immune cells generated by cell fate reprogramming to reach patients.

NK cells can be differentiated from human iPSCs using a two-stage protocol without additional sorting or the use of xenogeneic feeder cells. In this way, iPSCs are first instructed into hematopoietic progenitors and then stimulated with IL-3, IL-7, IL-15, SCF, and FLT3L to further promote NK differentiation. At the end of a 30-day culture period, induced NK cells expressed inhibitory and activating receptors including KIR, CD16, NKG2D, or NKp46 (151). Recently, Dege et al. identified iPSC-derived erythro-myeloid progenitors-like NK cells that are more potent in terms of cytotoxicity than adult CD16+ NK cells. Despite using the same protocol to induce NK cells, hematopoietic progenitors that resemble yolk sack precursors gave rise to NK cells with higher degranulation response when stimulated with K562 cancer cells, suggesting that this new population has potential for cancer immunotherapy (152). In addition, protocols to expand NK cells by the co-culture with artificial antigen-presenting cells such as K562 expressing membrane-bound 4-1BBL, IL-15, or IL-21 have been developed (153, 154).

Anti-tumor activity of induced NK cells can be further potentiated with a non-cleavable CD16a activating receptor which confers increased antibody-dependent cellular toxicity (155). In terms of function, in an ovarian xenograft mouse model, iPSC-NK cells reduced tumor burden comparably to NK cells purified from peripheral blood. Furthermore, iPSC-derived NKs may serve as a platform allowing modification of NK cells with CARs to improve cytotoxicity against NK therapy-resistant tumors. In this regard, Ueda et al. developed a feeder-free differentiation protocol employing FLT3L, IL-7, and phytohemagglutinin (PHA) to yield CAR-expressing NK/ILC cells from an iPSC line transduced with anti-GPC3-CAR. Notably, CAR NK/ILC were capable to elicit cytotoxic response against GPC3 expressing tumor cells, and prolonged survival when injected into mice with ovarian cancer (156). While the transcriptional code for direct reprogramming of NK cells has not been unveiled, Li et al. have provided evidence for the transdifferentiation of mouse T-cells into NK cells by the deletion of Bcl11b, a transcription factor that acts as a guardian of T-cell development and identity maintenance, allowing a rapid switch to NK identity (157).

### Reprogramming Into T-Cells

There is wide interest in utilizing iPSC technology towards the generation of naïve T-cells, as patient-derived T-cells are frequently rare or functionally exhausted. Large-scale, standardized production of cytotoxic T-cells would have an immense impact on CAR T-cell manufacturing. These systems are still under development to recapitulate fully functional T-cells and require extensive cell culture steps with cytokines or artificial thymic organoids (ATO) (158). The iPSC differentiation process itself recapitulates the natural T-cell development, transiting through hematopoietic precursors, double-positive CD4+CD8+ thymocytes, and maturation of CD4+ or CD8+ cells upon culturing with FLT3L and IL-7 on OP9-DL1 feeder layers mimicking NOTCH-ligand rich thymic microenvironment. Although initial studies failed to produce T-cells with a high level of resemblance to their natural counterparts, CD8+ T-cells generated from iPSCs elicited specific cytotoxic responses, despite some phenotypic differences including the absence of CD28 (159, 160). However, the maturation into CD4+ T-cells is even more challenging and requires ATO-based T-cell differentiation supported by FLT3L, SCF, and IL-7 (161). In an effort to move T-cell reprogramming further towards clinical applications, Iriguchi et al. developed a clinically applicable T-cell differentiation technique based on SDF1α and p38 inhibitor, allowing differentiation without feeder layers (162). For the specification of T-cells, the somatic origin of iPSC significantly impacts differentiation. iPSCs derived from cells of hematopoietic origin are more efficient to produce CD4+CD8+ double-positive lymphoid precursors, probably due to partially retained epigenetic memory which alleviates differentiation blocks in T-cell development (160). Moreover, iPSC derived from T-cells confer parental genetically rearranged TCR *loci* which will remain unchanged during reprogramming and differentiation, allowing the generation of rejuvenated T-cells that maintain antigen specificity (159, 160). In this regard, taking advantage of pre-existing anti-tumor antigen specificity, tumor-specific T-cells clones were reprogrammed to iPSCs to produce rejuvenated and functionally competent T-cells recognizing common tumor-specific antigens including MART-1 (melanoma), WT1 (leukemia) or GPC3 (hepatic and ovarian carcinoma) (159, 163). In regards to additional T-cell populations, Themeli et al. differentiated CAR-modified T-iPSC with FLT3L, SCF, IL-7 into CD3^+^TCR^+^CD8α^+^ CAR T-cells resembling γδ T-cells in their transcriptomic profiles and functions, and demonstrated that despite expressing endogenous αβ TCR, the induced T-cells conferred functional characteristics of innate lymphocytes (164).

While direct reprogramming of T-cells has not been reported, Zhang et al. showed the *in vivo* conversion of pro-pre-B cells into functional early T-cell lineage progenitors by transient expression of HOXB5. Although the conversion occurred in the bone marrow, early T-cell lineage progenitors migrate to the thymus and mature into functional polyclonal T-cells (165). The forward reprogramming approach has also been attempted by Guo et al., reporting a generation of T-cells from iPSCs *in vivo*. In this protocol, hematopoietic progenitors with thymus-homing abilities were generated by the expression of RUNX1 and HOXA9 at the endothelial-to-hematopoietic transition stage. Upon engraftment, these induced progenitor cells gave rise to T-cells that restore immune surveillance in immune-deficient mice (166).

Reprogramming within the lymphocyte lineage has also been shown to occur in humans. Interestingly, mutations in FOXP3, a transcription factor tightly controlling Treg homeostasis, functionally reprogram Treg towards Th2-like phenotype, causing immune dysregulation, and driving autoimmune polyendocrinopathy (167).

An additional approach to T-cell reprogramming relies on de-differentiation. Several publications demonstrated functional reprogramming of T-cells into stem cell-like memory CD8+ T-cells by inducing metabolic changes or the introduction of transcription factors. Memory T-cells self-renew, live longer and exert stronger effector functions, and therefore can act as a reservoir for effector T-cells with a potent therapeutic potential. Lu et al. promoted reprogramming of terminally differentiated effector T-cells into a less differentiated state *via* the overexpression of early T-cell differentiation-specific transcription factors LEF1, KLF7, ID3, EOMES, BCL6, TCF7, FOXP1, and FOXO1 (168).

Another type of reprogramming that does not entail a change in cellular identity is metabolic reprogramming. While it is well described that cancer cells undergo metabolic reprogramming to enhance survival in the hypoxic tumor environment (169), there is a growing body of evidence that metabolic alterations also occur in immune cells altering their function. Changes in the metabolic profile impacts differentiation and the activity of T-cells and other immune cells, a topic recently discussed by others (170, 171). In lymphocytes, metabolic reprogramming following T-cell activation imprints different functional fates. Interestingly, the promotion of lipolysis along with the inhibition of glycolysis induced by PD-1/CTLA-4 inhibition hamper T-cell effector differentiation (172). Zhang et al. hinted that increasing fatty acid oxidation in CD8+ T-cells prevents T-cell functional exhaustion and works synergistically with checkpoint inhibitors to delay or prevent tumor growth in murine cancer models (173). Exhausted tumor-infiltrating CD8+ T-cells were recently reinvigorated by IL-10 which revived their proliferative capacity and cytotoxic functions against cancer cells (174). Moreover, Verma et al. demonstrated reprogramming of CD8+ T-cells into memory stem cells with enhanced functional capacities using a MEK1/2 inhibitor, enhancing respiratory capacity and fatty acid oxidation, metabolic features previously associated with memory cells. Functionally, MEK inhibition administered with vaccination against known antigens (gp100 in melanoma, HPV16E7 in cervical carcinoma model) in murine cancer models successfully increased numbers of circulating CD8+ T-cells, reduced tumor burden, and improved survival rates (175). Indeed, manipulations of T-cell metabolism may help to improve the efficacy of T-cell-based immunotherapies (176).

### Reprogramming Into B-Cells

Generation of B-cells from iPSCs is equally challenging. Under specific conditions promoting lymphopoiesis, iPSCs can differentiate towards the B-cell lineage and form pre-B cells expressing CD45, CD19, and CD10. Reprogrammed B-cells upregulated the expression of pre-B specific genes including PAX5, IL7αR, and VpreB receptor. Interestingly, while they remained negative for surface IgM, induced cells displayed genomic D-J rearrangements, consistently with the adoption of pre-B identity (177). French et al. took the differentiation a step further and reported the generation of CD19+ CD10+ B lymphocytes *via* terminal differentiation with FLT3L, SCF, IL-3, and IL-7 on MS-5 stroma. iPSC-derived B-cells underwent full VDJ rearrangement and expressed surface IgM (178). While efforts in reprogramming and programming individual immune cell identities with fidelity will provide valuable cell sources for cancer immunotherapy, additional approaches take advantage of cellular reprogramming principles to modify cancer cell fate directly.

## Modifying the Cell Fate of Cancer Cells

Tumor development is accompanied by both epigenetic and genetic changes of the genome. Unlike genetic changes, epigenetic changes do not alter the primary DNA sequence and are therefore reversible. So, reprogramming paradigms are also highly relevant to understand oncogenesis, from the initial cellular transformation to the hierarchical organization of established malignancies (179). Indeed, alike somatic cells, cancer cells may be endowed with similar epigenetic flexibility. During reprogramming, the whole epigenetic landscape undergoes massive remodeling, and changes in histone modification and DNA methylation patterns underlie dramatic changes in gene expression (179). It was quickly hypothesized that the fate of cancer cells could be modified by direct reprogramming leading to a potential loss of tumorigenicity. The concept of reversing tumorigenicity dates to 1969, when experiments performed by Henry Harris attenuated malignant phenotype by fusing mouse cancer cells with fibroblasts, providing first insights into the activity of tumor suppressor genes (180). Experiments with somatic cell nuclear transfer – where melanoma nuclei were fused to enucleated oocytes showed that it is possible to re-establish pluripotency in cancer cells leading to differentiation into multiple benign cell types. Therefore, these experiments demonstrated that genetic and epigenetic changes of a melanoma nucleus are compatible with broad developmental potential but predispose mice to melanomas (181).

Reprogramming the cancer cell phenotype with transcription factors draws two relevant implications. First, induced pluripotent cancer stem cells are offering a valuable model to study cancer heterogeneity, cancer stem cells, and their genetic drivers as iPSC clones derived from single cells retain mutational signatures (182). Second, the reorganization of cancer cell epigenome may allow cancer cells to acquire a benign phenotype (183).

**Figure 3:**
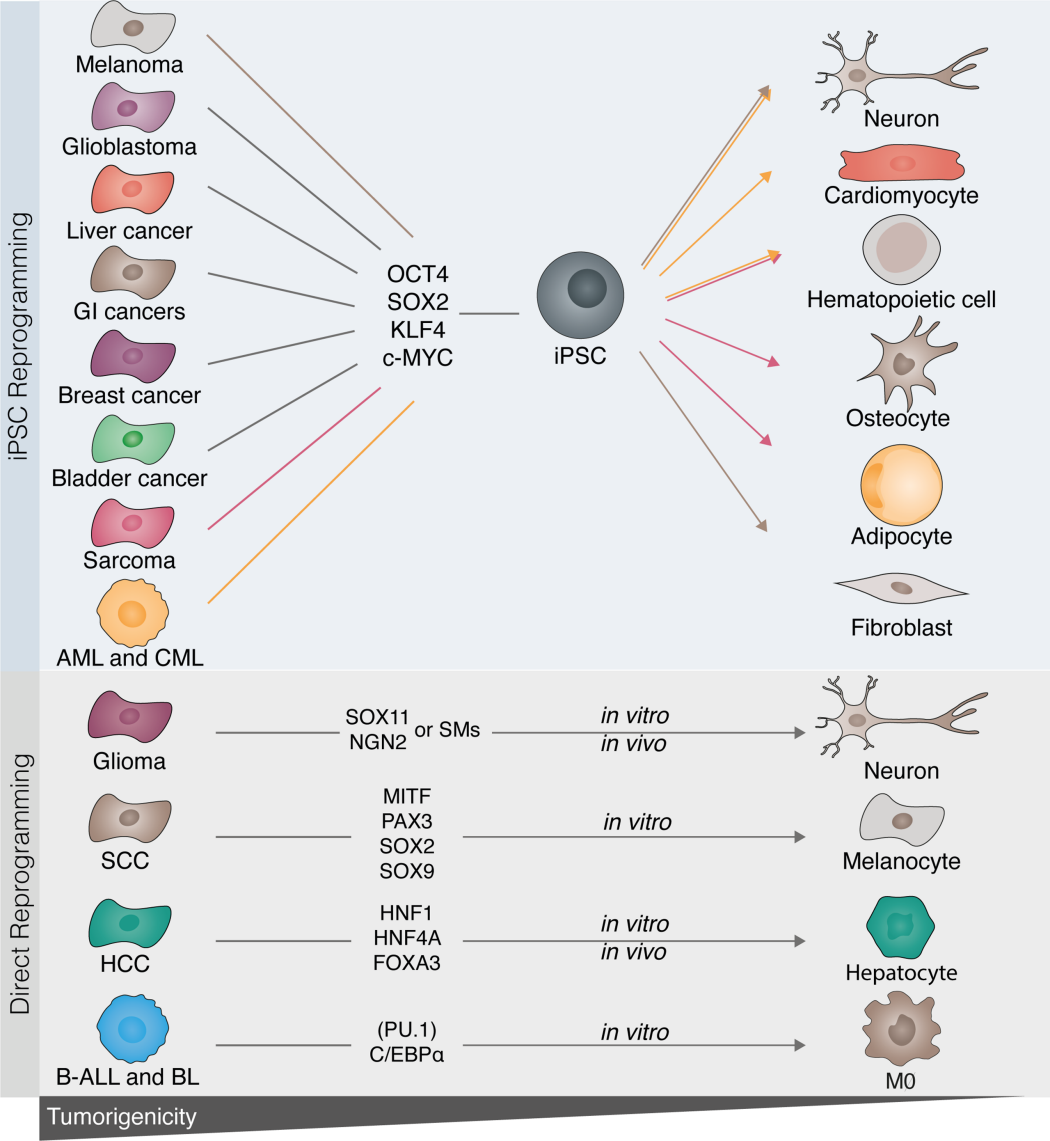
Disrupting cancer cell tumorigenicity with cellular reprogramming. Several tumor cell types have been described to be amenable to cell reprogramming towards induced pluripotent stem cells (iPSCs). Melanoma, glioblastoma, sarcoma, gastrointestinal (GI), as well as breast, bladder, and liver cancers, acute myeloid leukemia (AML), and chronic myelogenous leukemia (CML) have all been successfully reprogrammed towards iPSC with OCT4, SOX2, KLF4, and c-MYC. Following reprogramming to iPSCs, cancer cells can be differentiated into benignity by either differentiation to their original cell type or by converting them into a different lineage (highlighted by the colored lines). Similarly, *in vitro* direct reprogramming has been demonstrated in glioma, B-cell acute leukemia (B-ALL), Burkitt lymphoma (BL), squamous cell carcinoma (SCC), and hepatic cellular carcinoma (HCC). Lineage-specific factors rewrite the cancerous epigenetic and transcriptional program into mature terminally differentiated cells (glioma to neuron; SCC to melanocyte; HCC to hepatocyte), or by modifying cell fate and therefore disrupting the tumorigenic drive [B-leukemias to macrophage (MØ)].

Reprogramming of tumor cells into pluripotency was achieved in various types of malignant cells, however, given pre-existing reprogramming barriers, the efficiency remains low (184). Enforced expression of all or several of OSKM transcription factors generated iPSCs from multiple cancer cell lines including mouse melanoma (185), glioblastoma (186, 187), chronic myeloid leukemia (188), bladder cancer (189), breast carcinoma (190), and a range of gastrointestinal cancers (191) (**Figure 3**). Moreover, iPSC generation was achieved also from primary malignant leukemic blasts (192). In accordance with epigenetic rewiring, cancer reprogramming alleviated tumorigenicity *in vitro* and *in vivo* in most reprogrammed cells, even in those harboring constitutively active oncogenes. For instance, iPSC derived from BRAF mutated melanoma were further differentiated into non-tumorigenic neurons and fibroblasts displaying normal phenotype and functions (193). Furthermore, iPSC generated from primary BCR-ABL+ myeloid blasts differentiated into hematopoietic cells which did not retain leukemia potential. Upon transplantation, derived hematopoietic cells engrafted only temporarily and recipient animals did not develop chronic myeloid phenotype (192). Zhang et al. showed that sarcoma-derived iPSC generated primary connective tissue cells and erythroid cells. Moreover, when transplanted into mice, iPSC-derived adipocytes and osteoblasts did not form tumors, suggesting that reprogramming overrode tumorigenicity of parental sarcoma lines (183).

Interestingly, iPSC derived from MLL-rearranged acute myeloid leukemia cells could be differentiated into non-tumorigenic cardiomyocytes and neurons exerting physiologic functions despite the presence of oncogenic drivers. However, hematopoietic differentiation of AML-iPSC resulted in retrieval of leukemia potential (194).

### Direct Reprogramming of Cancer Cells Into Benignity

In contrast to the strategies forcing pluripotency in cancer cells, direct reprogramming allows the modification of somatic identity and may provide an undeviating way to disrupt the epigenetic tumorigenic drive. Considering the impact of CEBPα on reprogramming of B-cells into the myeloid lineage, Rapino et al. utilized its enforced expression to induce macrophage identity in human B-cell leukemia and lymphoma, generating quiescent phagocytic cells with transcriptomic profiles resembling macrophages (**Figure 3**). Importantly, activation of macrophage regulatory network, including endogenously expressed CEBPα, sustained the newly acquired macrophage identity (195). Similarly, McClellan et al. transdifferentiated primary acute leukemia blasts into macrophages either with overexpression of CEBPα and PU.1 or upon exposure to a myeloid cytokine cocktail comprising FLT3L, IL-7, IL-3, GM-CSF, and M-CSF, overcoming the differentiation block and reducing leukemogenicity (196).

Loss of tumorigenicity is also induced when cancer cells from the same lineage are induced into mature differentiated cells. Melanocytes induced from squamous cell carcinoma *via* overexpression of MITF, PAX3, SOX2, and SOX9 lose tumorigenicity, as shown by reduced proliferation, migration, and invasion (197). To explore enforced differentiation in the context of liver cancer, Cheng et al. deployed a combination of transcription factors HNF1A, HNF4A, and FOXA3 to reprogram hepatocellular carcinoma into hepatocytes. Importantly, reprogrammed hepatocytes retrieved benign functions including albumin and glycogen production, and actively contributed to liver regeneration in a murine model of genetic liver dysfunction (113). In glioblastoma, NGN2 and SOX11 were employed to force post-mitotic arrest and differentiation of glioma into neurons (198). In an attempt to develop transgene-free reprogramming methods, neuronal reprogramming in glioma was recently induced with ROCK and mTOR inhibitors (199). Alternatively, Yuan et al. induced a similar conversion with a small compound cocktail composed of forskolin, ISX9, DAPT, CHIR99021, and I-BET 151 (200).

Another solution that does not strictly reflect classical reprogramming was recently proposed by Ishay-Ronan et al. Exploiting the brief window of high cellular plasticity during epithelial-mesenchymal transition (EMT), breast carcinoma cells were diverted into post-mitotic functional adipocytes with a cocktail composed of TGF-β, BMP2, insulin, and dexametazone. Notably, rosiglitazone-induced EMT together with adipogenic differentiation therapy efficiently prevented cancer cell invasion and formation of metastasis *in vivo* (201). However, not all conversions of cellular identity entail the loss of tumorigenicity. Reprogramming of primary Pax^+/-^Ebf^+/-^ pro-B leukemic cells into the T-cell lineage *via* constitutive induction of NOTCH1 forced lineage conversion into early-stage T-cells which displayed leukemic phenotype and potential (202).

### Inducing Antigen Presentation in Cancer Cells with Cellular Reprogramming

A large body of evidence indicates that inhibition of antigen presentation is one of the major mechanisms underlying cancer immune evasion, allowing tumor cells to become invisible to immune effector cells. Therefore, restoration or enforcement of antigen presentation machinery in cancer cells holds enormous potential to reactivate cytotoxic anti-tumor responses. While all cell types can present their own antigens through HLA class I molecules, only professional antigen-presenting cells, such as DCs or macrophages, can prime productive T-cell responses by providing costimulatory signals besides HLA class I/II and TCR interactions. Interestingly, expression of class I and class II molecules in tumor cells can be, at least to a certain extent, induced by radiotherapy, several chemotherapeutic agents, a range of small compounds including histone deacetylase (HDAC) inhibitors, tyrosine kinase inhibitors (203), CDK4/6 inhibitors (204), or IFN-γ (205). Unfortunately, instead of boosting immune surveillance, these compounds may further escalate immunosuppression through induction of T-cell anergy triggered by TCR engagement in the absence of proper co-stimulation. In this context, reprogramming of cancer cells towards professional antigen-presenting cells may represent an unexplored avenue to elicit anti-tumor polyclonal T-cell responses. The establishment of dendritic-like cell identity in tumor cells could aid immune surveillance reactivation in a more complex way, removing two critical barriers of efficient anti-tumor immunity – deficient antigen presentation of tumor antigens and insufficient T-cell activation. Moreover, as DC-reprogrammed tumor cells could promote the recruitment of T-cells to the tumor site by the secretion of CXCL9 and CXCL10, such strategy could also potentiate the effect of other T-cell-based cancer immunotherapies including immune checkpoint blockade and adoptive cell transfer.

## Reprogramming *In Vivo*

### Inducing Cell Fate Reprogramming *In Vivo*

**Figure 4:**
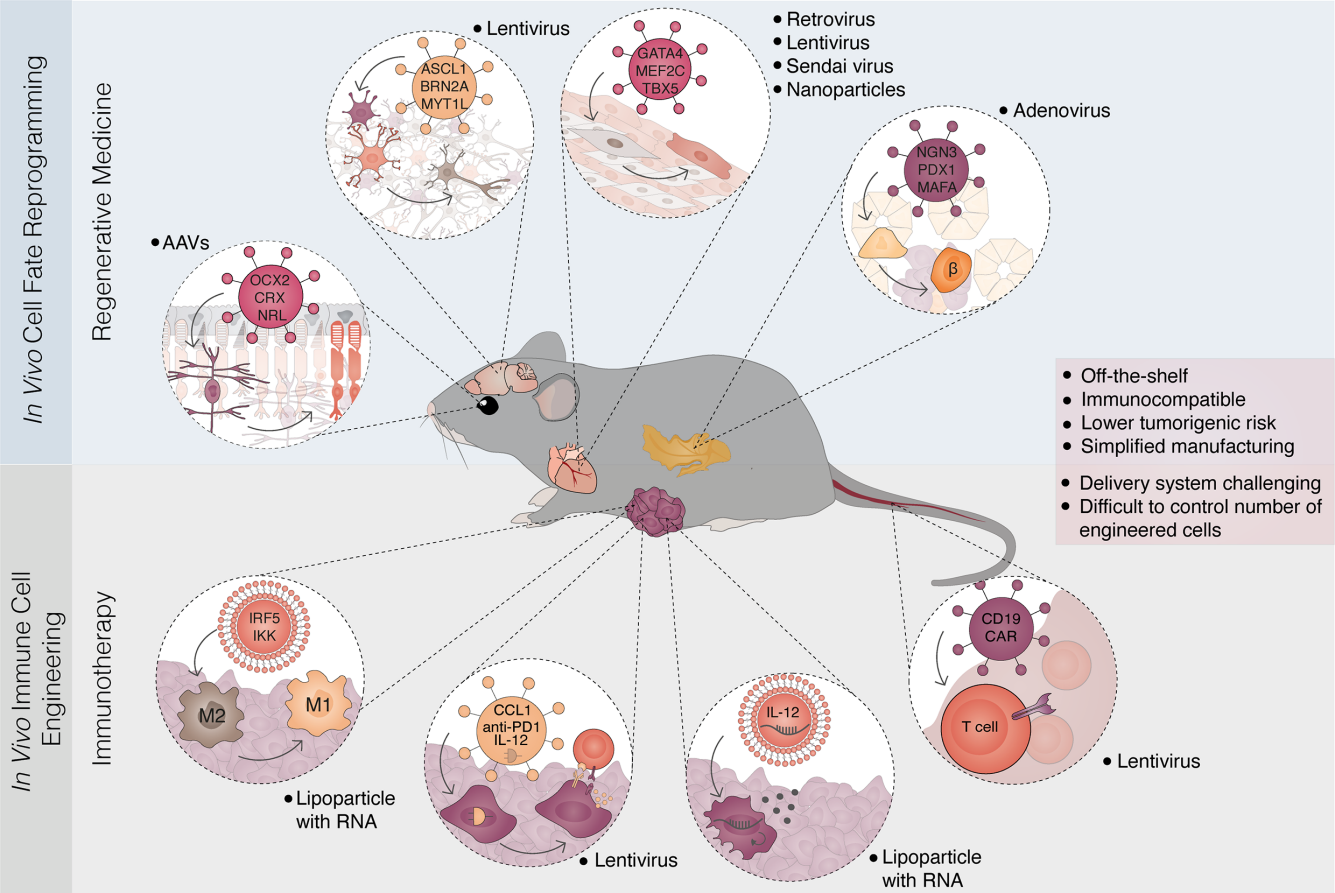
*In vivo* reprogramming strategies for regenerative medicine and immunotherapy. Overview of parallel *in vivo* approaches for cell fate reprogramming and immune cell engineering. *In vivo* cell fate reprogramming offers an approach to restore damaged cell types and tissue function (top panel). The concept has been demonstrated in multiple tissues and cellular conversions. Examples include the reprogramming of Müller glia cells to photoreceptors leading to restoration of vision in congenitally blind mice; reprogramming of astrocytes and glial cells to neurons; the conversion of fibrotic tissue after myocardial infarction to functional cardiomyocytes and the reprogramming of pancreatic acinar/exocrine cells into insulin-producing β-cells. These strategies mainly rely on the delivery of lineage-specific factors through viral vectors. In parallel, *in vivo* engineering of immune cells has been developed allowing the generation of genetically modified immune cells, often designated immune reprogramming (bottom panel). Recent examples in cancer immunotherapy include the conversion from macrophage type 2 (M2) to macrophage type 1 (M1), manipulation of cancer cells to express immunogenic circuitry and cytokines, and the *in vivo* delivery of chimeric antigen receptors (CAR) to circulating T-cells. Delivery strategies are diverse including lipid nanoparticles coupled with DNA or replicative RNA, and viral vectors (lentivirus, AAV, retrovirus, adenovirus, Sendai virus). *In vivo* approaches in regenerative medicine and immunotherapy converge towards the creation of off-the-shelf, immunocompatible therapies that harbor low tumorigenic risks. Simplified clinical translation and manufacturing are balanced by challenges in delivery and the control of cell-type specificity and numbers. AAV, adeno-associated virus.

iPSC differentiation and direct reprogramming are now starting to provide the means to address the potential for cancer immunotherapy of unique patient-tailored immune cell types. However, long-term survival and functional integration within the target tissue remain long-term challenges for transplantation of *in vitro* generated cells. In parallel with *ex vivo* based therapy, the development of viral vectors for gene delivery has opened opportunities for *in vivo* reprogramming in regenerative medicine, an elegant strategy circumventing current limitations of *in vitro* cell manipulation. Therefore, the possibility of switching cell identity within the living organism gained attention for its immense therapeutic potential. The main advantages of inducing immune cell reprogramming *in vivo* would be the use of internal cell sources, limited immune rejection, limited risk for tumor formation and increased reprogramming efficiency and fidelity (206). In an *in vivo* setting, cells receive a full set of external cues that are difficult to recapitulate *in vitro*, maximizing the efficiency of reprogramming. However, efficient, safe, cell-specific delivery of transcription factors required for eliciting reprogramming represents the major bottleneck (**Figure 4**). Safety concerns accelerated the development of non-integrative viral vectors, non-viral delivery systems, as well as prompt efforts, and the design of cell type-specific promoters for more precise targeting (207–211).

*In vivo* reprogramming has been demonstrated with the expression of OCT4, SOX2, KLF4, and c-MYC and the induction of pluripotency. These studies demonstrated the feasibility of inducing transcription factor-mediated reprogramming *in vivo* but also highlighted the role of the tissue microenvironment. As the microenvironment and the presence of internal cues have a profound effect on cellular plasticity, local factors significantly impact the efficiency of the reprogramming process. Two independent studies highlighted the favorable effect of senescence, which *in vitro* significantly hampers reprogramming. Aging or cell damage triggers senescence and induces secretion of inflammatory cytokines, enhancing the natural plasticity of neighboring cells, and making them more amenable to undergo lineage conversion. Notably, lung epithelial cells could have been generated *in vivo* only after bleomycin-induced damage, accentuating the impact of injury regenerative response on intrinsic cell plasticity (212, 213).

Direct cell reprogramming between unrelated somatic cells holds more attractive therapeutic prospects. Injury response may lead to fibrotic tissue damage and loss of function, as seen for instance after myocardial infarction or brain injury. In this context, *in vivo* direct reprogramming offers an interesting alternative to restore damaged cell types and tissue function (**Figure 4**). In mice, resident cardiac fibroblasts were reprogrammed into functional cardiomyocytes with GATA4, MEF2C, and TBX5, counteracting adverse ventricular remodeling and improving function after a heart attack (112, 214). Similarly, overexpression of neural transcription factors in astrocytes and glial cells triggered reprogramming into neurons that were able to integrate within the existing neuronal circuits (215–217). Furthermore, encouraging results were delivered by conversion of pancreatic acinar/exocrine cells into insulin-producing β-cells (120, 218) or by induction of functional hepatocytes in the murine fibrotic liver, leading to an improvement in liver performance (219, 220). Notably, restoration of liver function was achieved also with hepatocytes reprogrammed *in vivo* from hepatocellular carcinoma cells (113). More recently, Kurita et al. turned wound resident mesenchymal cells into epithelial skin cells with DNP63A, GRHL2, TFAP2A, and MYC (221), while Yao et al. utilized *in vivo* reprogramming to restore vision in congenitally blind mice, with the overexpression of OCX2, CRX, and NRL. This transcription factor combination promoted photoreceptor identity in proliferating Müller glia cells preconditioned with β-catenin (222). Despite the numerous examples and vast range of applications in regenerative medicine, *in vivo* reprogramming paradigms have not yet been explored for immunotherapy.

### *In Vivo* Immune Cell Engineering

An analogous approach has been recently developed in cancer immunotherapy by the generation of immune cells engineered *in vivo*. Thus, there is a conceptual parallel between *in vivo* cell fate reprogramming and *in vivo* immune genetic engineering (often named immune reprogramming), highlighting a possible convergence of approaches towards cancer immunotherapy (**Figure 4**). The engineering of immune cells may help to overcome cancer therapy limitations, including lack of targetable antigens or tumor-mediated immune suppression. Although immune-stimulatory factors such as cytokines and chemokines could be directly used to attempt to overcome these challenges, systemic administration often results in strong off-target activity and severe adverse effects, underscoring the need for *in situ* solutions.

The maintenance of tolerogenic tumor niche is attributed to tumor-associated macrophages (TAM). Their targeted elimination *via* CSF1R blockade emerged as a potential solution to reactivate localized immune responses (223). However, although CSF1R inhibition limited survival of TAMs and promoted favorable TAM polarization and elimination of glioblastoma in mice (224), also impacted tissue-resident macrophages and brought systemic toxicity. Interestingly, the mission to prevent pro-tumorigenic activities of TAMs may be also accomplished with the reprogramming of M2-polarized TAMs towards M1 pro-inflammatory phenotype. Exploiting the intrinsic phagocytic ability of macrophages, Zhang et al. reprogrammed TAMs with nanoparticles delivering M1 polarizing factors IRF5 and IKK and showed, that repeated delivery of therapeutic lipoparticles abrogated tumor growth and significantly prolonged survival in mice with ovarian carcinoma, melanoma, and glioblastoma. Notably, surviving mice did not form tumors when re-challenged with cancer cells, indicating that reprogramming-based macrophage polarization induced durable systemic immunity which may prevent the formation of metastasis (225).

The immunogenicity of cancer cells is another fundamental determinant of the immune response. The strategy to increase tumor immunogenicity reported by Wang et al. consisted of intratumoral delivery of activating CRISPRa components to augment endogenous expression of TAAs. In mouse melanoma and pancreatic adenocarcinoma models, CRISPRa-mediated enhanced expression of TAAs increased the proportion of antigen-presenting cells and T-lymphocytes within the tumor microenvironment and elicited systemic anti-tumor immunity. Notably, the developed platform can be modified to direct simultaneous activation of expression of multiple TAAs as well as other immunomodulatory molecules, chemokines, or cytokines to further support cancer immune responses (226). A strategy based on RNA was developed by Li et al. with an oncolytic nanoparticle-based system delivering self-replicating RNA encoding IL-12. The combination of immunogenic cell death with high levels of IL-12 elicited a systemic immune response and eradicated melanoma and colon carcinoma in mice (227). Similarly, functional engineering of tumors enhancing immunogenicity was recently accomplished by Tzeng et al. who used nanoparticles to transfect and overexpress 4-1BBL and IL-12 in cancer cells. Considering that most cancer cells retain at least low levels of HLA class I molecules displaying tumor antigens, additional expression of the costimulatory molecule and IL-12 may endow cancer cells with the ability to prime T-cells and trigger NK cell activation, mimicking actions of true professional antigen presenting cells. Notably, functionally reprogrammed cells elicited systemic cell mediated anti-tumor responses and in combination with checkpoint blockade significantly abrogated tumor growth in mice with melanoma and colorectal carcinoma, inducing systemic cancer immunity (228).

A riveting solution to boost tumor immunogenicity and to fuel local anti-cancer immune responses was recently suggested by Nissim et al. who engineered lentiviral-delivered synthetic gene circuits driving expression of immune-modulating components including a surface T-cell engager, the chemokine ligand CCL21, IL-12, and anti-PD1 antibody directly in tumor cells. To prevent unwanted toxicity and the risk of autoimmunity due to the off-target delivery into neighboring non-malignant cells, the expression of immune-modulating agents was linked to the activation of cancer-specific promoters, ensuring that the immune response is directed exclusively against cancer cells. Interestingly, the proposed immune-modulating therapy elicited a robust T-cell mediated response and resulted in the rejection of ovarian cancer (229).

### *In Vivo* Generation of CAR T-Cells

*Ex vivo* produced CAR T-cells recognizing tumor antigens has proved as a highly effective treatment for patients with refractory B-cell malignancies. The manufacturing process which requires extensive and time-consuming procedures is the limiting step. To simplify the process, Pfeiffer et al. recently developed lentiviral vectors enabling highly selective targeting of CD8+ cells which is used for *in vivo* generation of CAR T-cells. Despite the relatively low transduction efficiency (not exceeding 5%), *in vivo* engineered CD19-CAR T-cells demonstrated the ability to eliminate CD19+ cells in mice (230). Importantly, a single injection of CD8 targeted lentivirus was sufficient to generate CD19-CAR T-cells and efficiently eradicate CD19+ leukemic blasts in mice within two weeks (231). Elimination of malignant B-cells was achieved also with *in vivo* modified CD4+ T-cells expressing CD19-CAR within 2-3 weeks. Interestingly, in tumor-bearing mice, CD4+ CD19-CAR T-cells exhibited higher anti-CD19 activity and outperformed CD8+ CAR T-cells especially in the context of higher tumor burden, suggesting that CD4+ CAR T-cells are less prone to exhaustion (232). In the clinical context, *in vivo* CAR generation may have major implications on the manufacturing process and facilitate the development of off-the-shelf CAR T-cell therapy, making it accessible for a larger group of patients.

Cancer immunotherapy has revolutionized cancer treatment. The limits of the current strategies both individually or in combination will be elucidated in the coming years. Harnessing cell reprogramming concepts towards immunotherapy represent an entirely new set of approaches with the potential to take advantage of the full immune repertoire, their identity, and the individual immunity modules under their control. Possibilities range from novel sources of allogeneic immune cells, rejuvenating functionally exhausted effector cells, disrupting vicious regulatory networks of cancer cells, or modifying the immunosuppressive tumor microenvironment by inducing immune cell identities *in vivo*. Overall, the redefinition of immune cell identity and their regulatory circuits by cell fate reprogramming is now providing new and exciting tools for the fast-paced field of cancer immunotherapy.

## Author Contributions

OZ, IC, AGF, and C-FP conceptualized this manuscript. IC prepared the figures. AGF wrote figure legends. OZ and C-FP wrote the manuscript. All authors contributed to the article and approved the submitted version.

## Funding

This project was co-funded by Cancerfonden (20 0939 PjF), the Swedish research council (2020-00615), European Research Council (866448-TrojanDC), Novo Nordisk Foundation (0056527) and FCT (PTDC/MED-IMU/4520/2020). The Knut and Alice Wallenberg foundation, the Medical Faculty at Lund University and Region Skåne are acknowledged for generous financial support. OZ is supported by a Cancerfonden postdoctoral fellowship 190008. IC and AGF are supported by FCT doctoral fellowships (PD/BD/146424/2019 and SFRH/BD/133233/2017).

## Conflict of Interest

The authors declare that the research was conducted in the absence of any commercial or financial relationships that could be construed as a potential conflict of interest.
